# Is it psychosis? Heads or tails. A case report

**DOI:** 10.1192/j.eurpsy.2022.2016

**Published:** 2022-09-01

**Authors:** B. Rodado León, M. Huete Naval, A. García Carpintero, M. Jiménez Cabañas, A. Bermejo Pastor, M. Pérez Lombardo

**Affiliations:** 1 Hospital Clínico San Carlos, Instituto De Psiquiatría Y Salud Mental, Madrid, Spain; 2 Hospital Clínico San Carlos, Institute Of Psychiatry And Mental Health, Madrid, Spain; 3 Hospital Clínico San Carlos, Institute Of Psyquiatry And Mental Health, Madrid, Spain; 4 Hospital Clínico San Carlos, Psychiatry, Madrid, Spain

**Keywords:** schizophrénia, heavy metals, PSYCHOTIC DISORDERS, argyria

## Abstract

**Introduction:**

Psychotic disorders usually come with diagnosis difficulties, especially when the clinical presentation is recent or if there are organic factor associated. Regarding this, we propose the clinical case of a man 47 years old without psychiatric history, who is brought to the hospital after being run over by the subway. At his arrival, he verbalizes delirious thoughts of persecution and harm.

**Objectives:**

The objective is to emphasize the importance of making an appropriate somatic study in psychosis cases, especially when we don’t know the time of setting or we can’t make a psychiatric interview in optimal conditions.

**Methods:**

The study included a blood test including methemoglobine, cranial tomography, serologies and a heavy metals test. We reviewed the scientific literature in Pubmed and Web of Science about the possible association between the psychiatric and the dermatological symptoms.

**Results:**

During his admission, the patient recognizes delusional thoughts of harm since he was young and he was so frightened because of this that he tried to commit suicide in the subway. Moreover, he also thinks that silver can heal any disease, so he has licked silver coins for years. The final diagnosis was schizophrenia, and argyria due to a chronic silver intoxication.

**Conclusions:**

Heavy metals intoxications can be associated to acute psychotic disorders, so we must take them into account. As well, schizophrenia can cause bizarre believes which can lead to the intoxication.

**Disclosure:**

No significant relationships.

